# Supplementary material for: Adipocyte PHLPP2 inhibition prevents obesity-induced fatty liver
Source: Nat Commun. 2021 Mar 23;12:1822. doi: 10.1038/s41467-021-22106-2 (PMC7988046; doi:10.1038/s41467-021-22106-2)
Supplement: Supplementary file 1 — Supplementary Information [file 41467_2021_22106_MOESM1_ESM.pdf]

Supplementary Fig. 1

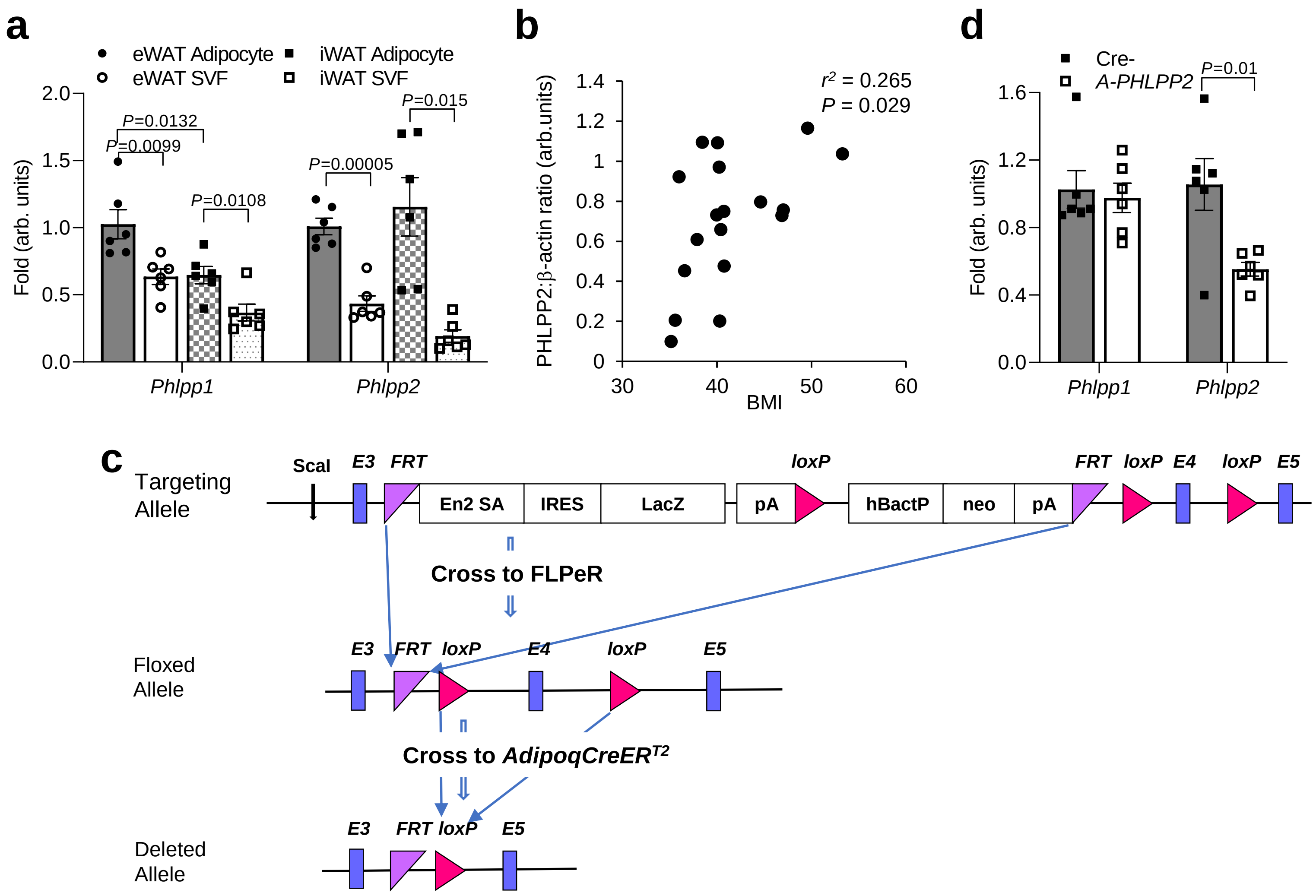

Supplementary Fig. 1 Generation of conditional *PHLPP2* KO mice. **a** *Phlpp1* or *2* gene expression in floated adipocytes and pelleted stromal vascular fraction (SVF) isolated from epididymal and inguinal (subcutaneous) WAT from C57BL/6J mice (n=6 independent mice per group). **b** Visceral adipose PHLPP2 levels are corelated to BMI, as analyzed by linear regression (n=18 per independent samples). **c** Targeting strategy to generate adipocyte-specific *PHLPP2* knockout (*A-PHLPP2*) mice. **d** *Phlpp1* or *2* gene expression in eWAT from Cre- and *A-PHLPP2* mice (n=6 independent mice per group). All data are shown as the means ± SEM. Statistical significance was determined by unpaired two-tailed Student's *t*-test.

Supplementary Fig. 2

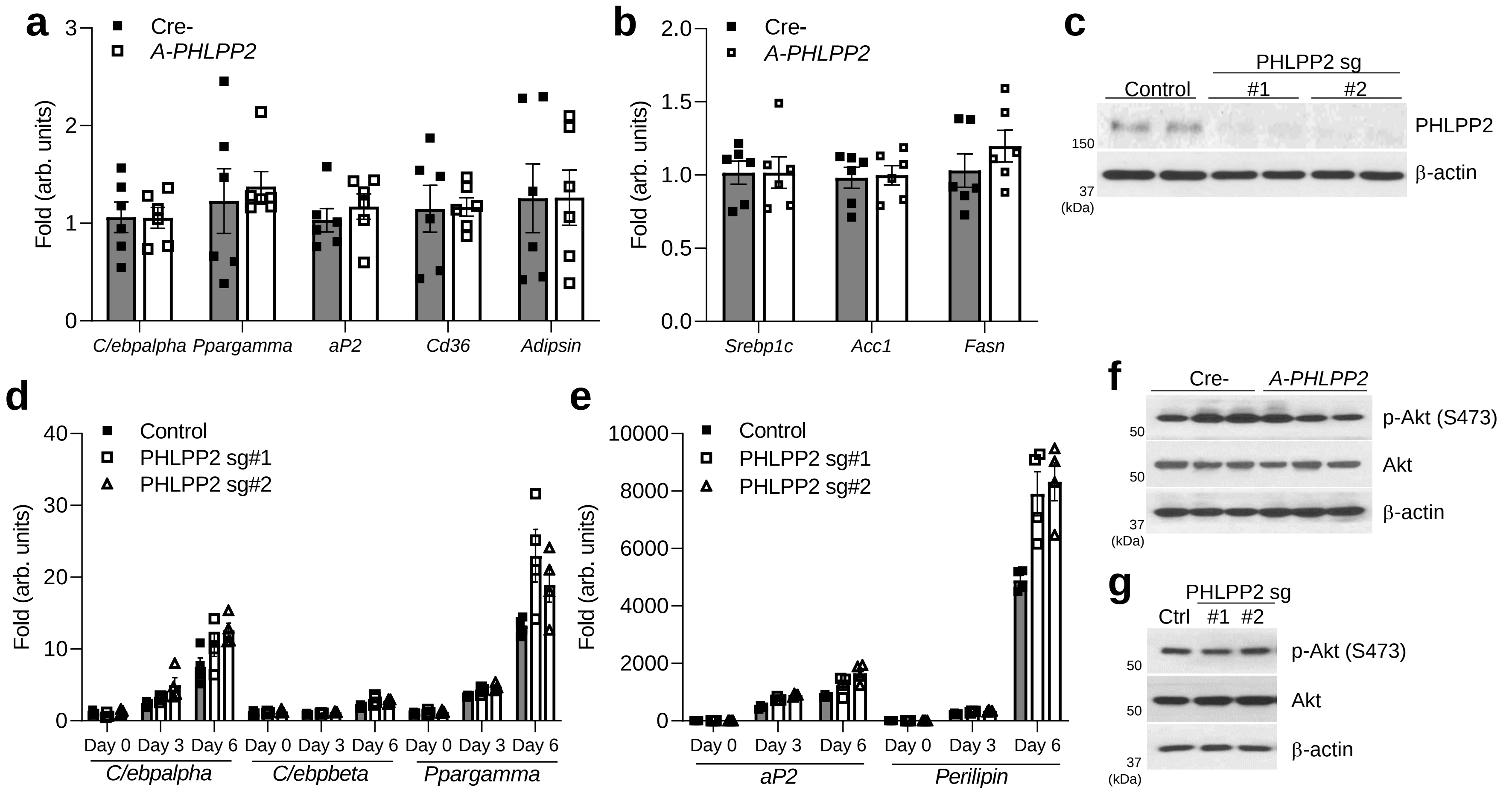

Supplementary Fig. 2 Adipogenic gene expression and Akt Ser473 phosphorylation are unaffected by PHLPP2 ablation. **a, b** Gene expression in eWAT from HFD-fed Cre- and *A-PHLPP2* mice (n=6 independent mice per group). **c-e** Western blot (**c**) or gene expression in differentiated control or PHLPP2-repressed (PHLPP2 sg) 3T3-L1 adipocytes (n=4 independent samples) (**d, e**). **f, g** Western blot in eWAT from HFD-fed Cre- and *A-PHLPP2* mice (n=3 independent mice per group) (**f**) or control of PHLPP2-repressed 3T3-L1 adipocytes (**g**). All data are shown as the means ± SEM.

Supplementary Fig. 3

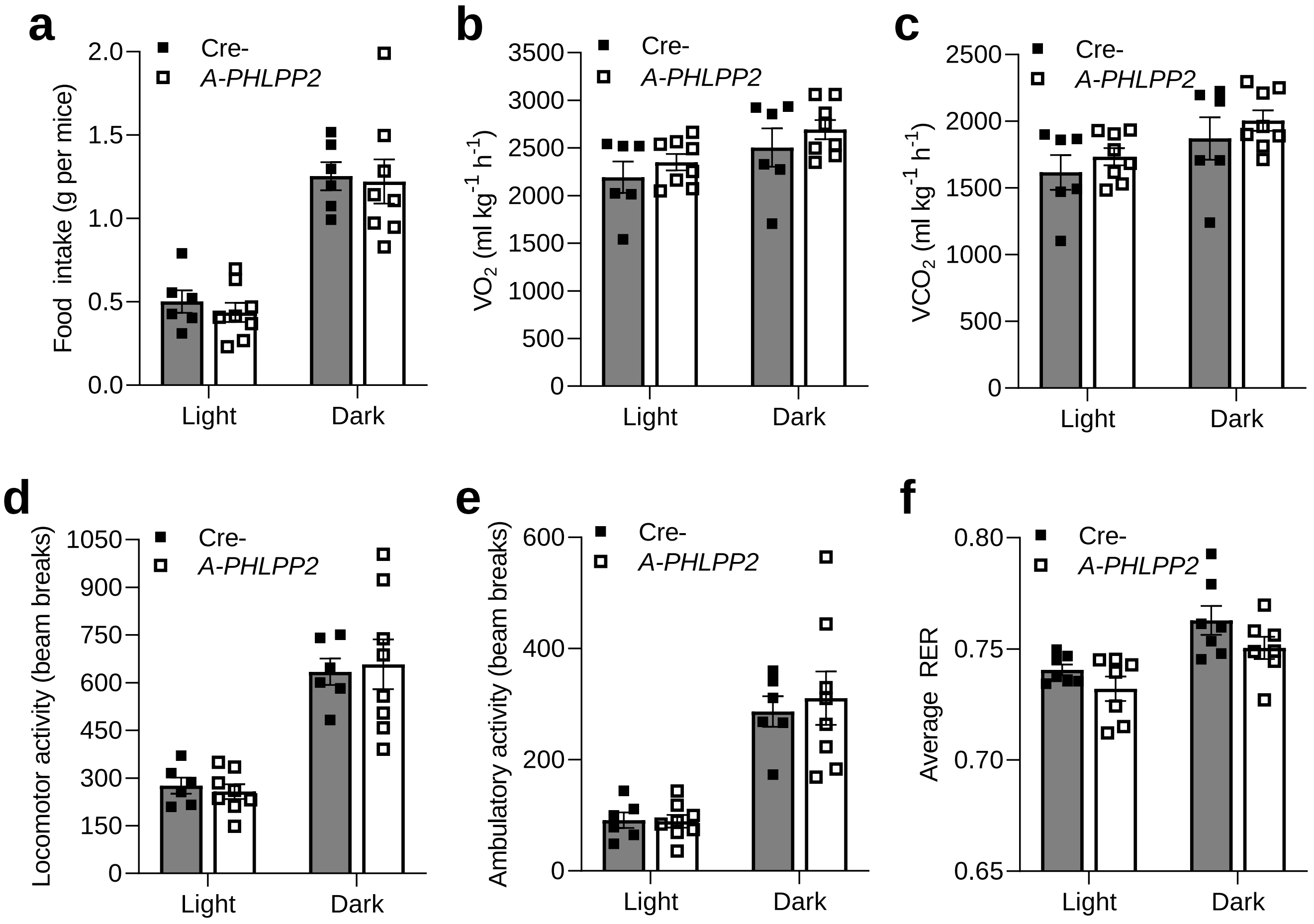

Supplementary Fig. 3 Energy expenditure effects of PHLPP2 ablation. **a-f** Food intake (**a**), and VO_2 (**b**), VCO_2 (**c**), locomotor activity (**d**) or ambulatory activity (**e**) in HFD-fed Cre- and *A-PHLPP2* mice (n=6-8 independent mice per group) or RER (**f**) in HFD-fed Cre- and *A-PHLPP2* mice with CL316,243 treatment (n=7 independent mice per group) as measured by indirect calorimetry. All data are shown as the means ± SEM.

Supplementary Fig. 4

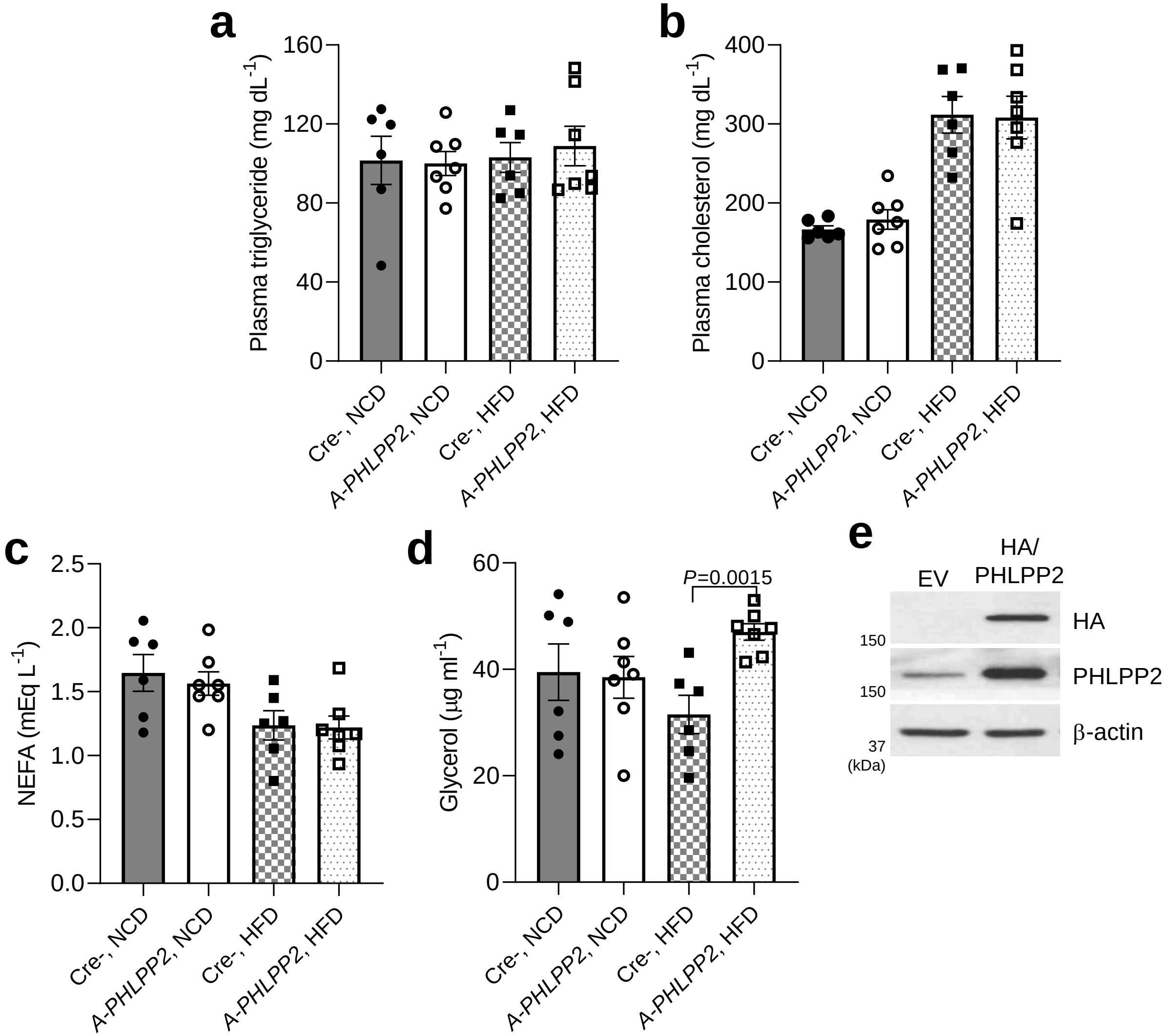

Supplementary Fig. 4 Metabolic analysis of *A-PHLPP2* mice. **a-d** Plasma triglyceride (**a**), cholesterol (**b**), NEFA (**c**), and glycerol (**d**) in NCD- or HFD-fed Cre- and *A-PHLPP2* mice (n=6-7 independent mice per group). **e** Western blots from differentiated 3T3-L1 adipocytes with PHLPP2 overexpression. All data are shown as the means ± SEM. Statistical significance was determined by unpaired two-tailed Student’s *t*-test.

Supplementary Fig. 5

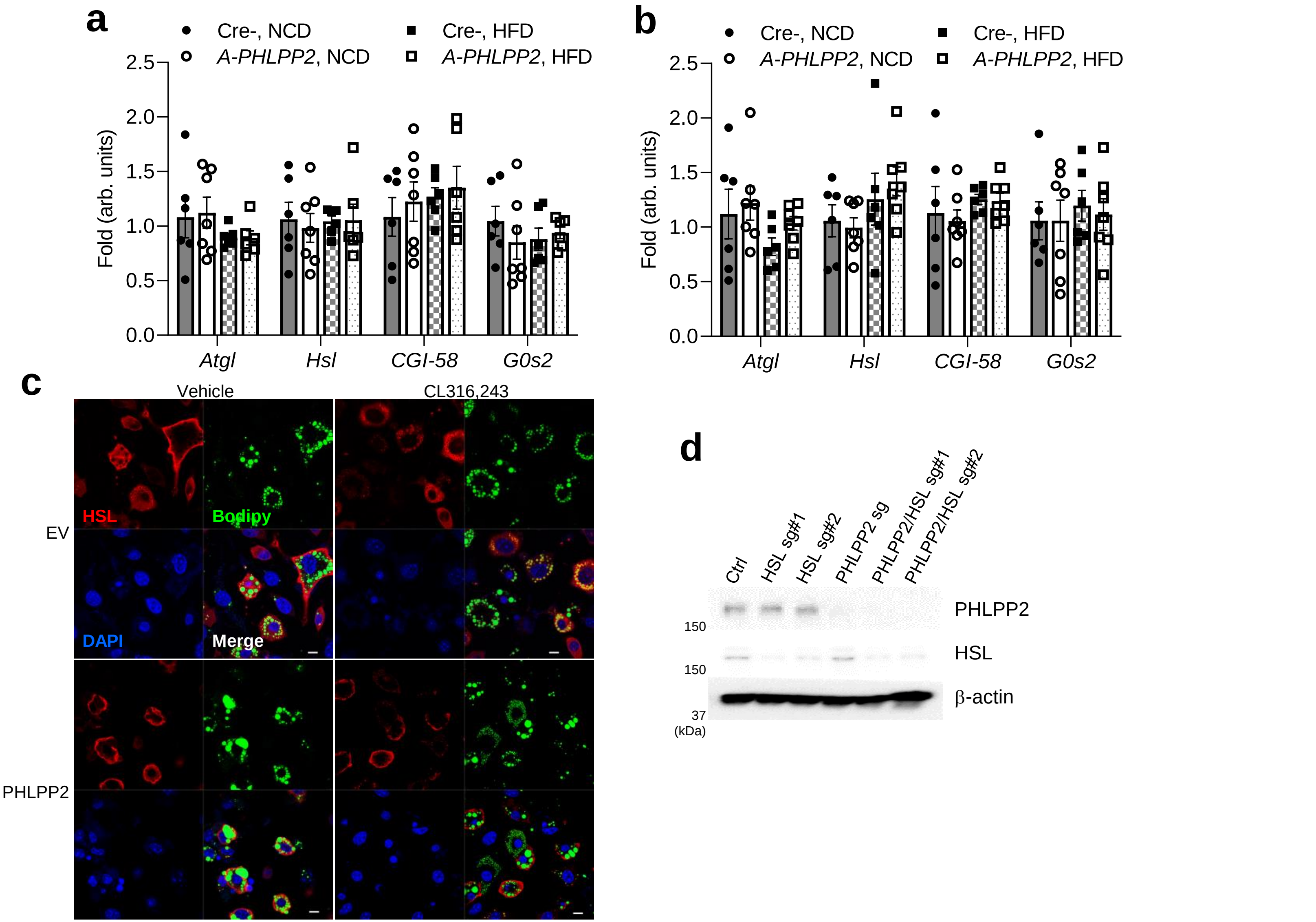

Supplementary Fig. 5 Lipolytic gene expression is unaffected by PHLPP2 ablation. **a, b** Expression of key lipolytic genes in eWAT (**a**) or iWAT (**b**) from NCD- or HFD-fed Cre- and *A-PHLPP2* mice (n=6-7 independent mice per group). **c** Additional immunofluorescence result of HSL (red) and Bodipy (green) in 3T3-L1 adipocytes expression control of HA-tagged PHLPP2 with or without CL316,243. Scale bar, 10 μm. **d** Western blots from differentiated 3T3-L1 adipocytes with control, *PHLPP2*, *HSL* and/or *PHLPP2/HS*L double repression. All data are shown as the means ± SEM.

Supplementary Fig. 6

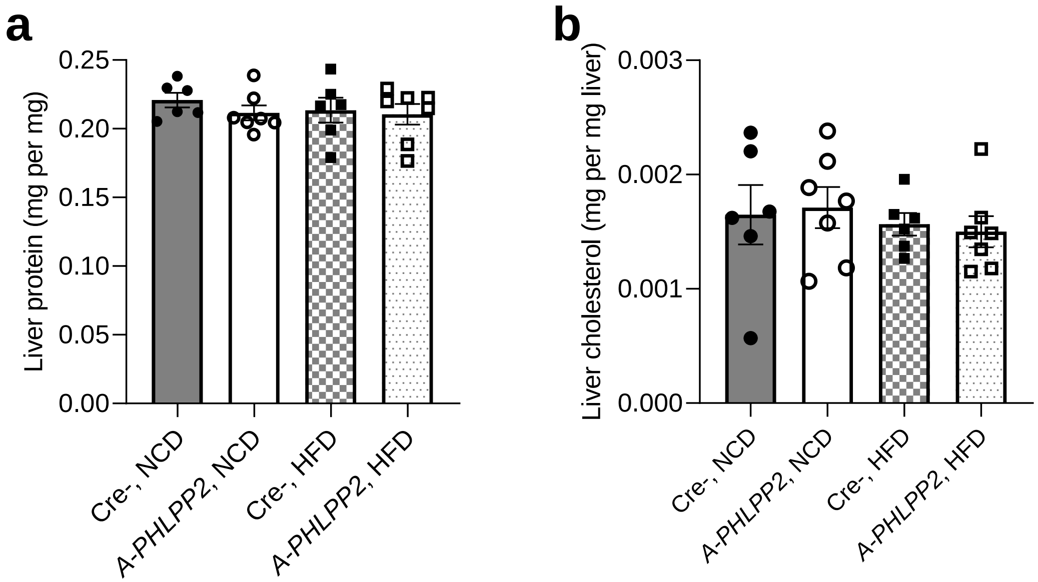

Supplementary Fig. 6 Liver effects of adipocyte PHLPP2 ablation. **a, b** Liver protein (**a**) and cholesterol (**b**) in HFD-fed Cre- and *A-PHLPP2* mice (n=6-7 independent mice per group). All data are shown as the means ± SEM.

Supplementary Fig. 7

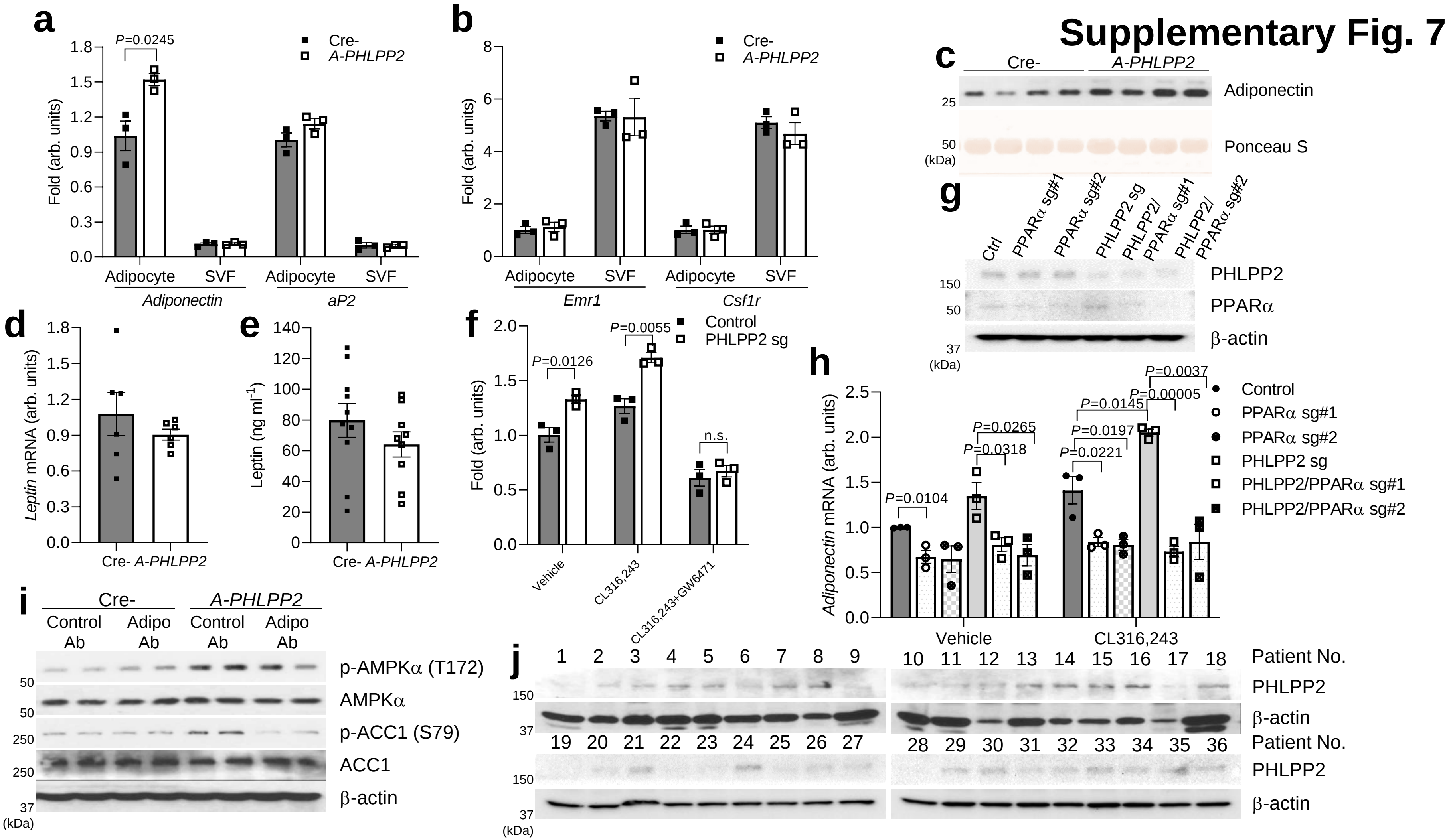

Supplementary Fig. 7 PHLPP2 affects adiponectin expression and levels. **a-c** Gene expression from isolated adipocytes and SVF from eWAT (n=3 independent mice per group) (**a, b**), and serum adiponectin (**c**) from HFD-fed Cre- and *A-PHLPP2* mice (n=4 independent mice per group). **d, e** *Leptin* mRNA expression in eWAT (n=6 independent mice per group) (**d**) and serum leptin (n=9-10 independent mice per group) (**e**) from HFD-fed Cre- and *A-PHLPP2* mice. **f** *Adipoq* expression from control or PHLPP2-repressed 3T3-L1 adipocytes, with or without CL316,243 and/or GW6471 (n=3 independent samples). **g, h** Western blots (**g**) and *Adipoq* expression (**h**) from differentiated 3T3-L1 adipocytes with control, *PHLPP2*, *PPARα* and/or *PHLPP2/PPARα* repression (n=3 independent samples). **i** Western blots from primary hepatocytes after treatment with serum from either Cre- or *A-PHLPP2* mice pre-neutralized with control or anti-Adiponectin (Adipo) Ab (n=2 independent samples). **j** Western blots of PHLPP2 and β-actin in lysates of adipose tissues from 36 human subjects. All data are shown as the means ± SEM. Statistical significance was determined by unpaired two-tailed Student's *t*-test.

Supplementary Table 1. Quantitative PCR primer sequences

Gene Symbol	Sequences (5' to 3')	
	Forward	Reverse
36b4	AGATGCAGCAGATCCGCAT	GTTCTTGCCCATCAGCACC
Acc1	AGCAGATCCGCAGCTTG	ACCTCTGCTCGCTGAGTGC
Acox	GTGCAGCTCAGAGTCTGTCCAA	TACTGCTGCGTCTGAAAATCCA
Adiponectin	GCACTGGCAAGTTCTACTGCAA	GTAGGTGAAGAGAACGGCCTTGT
Adipsin	CATGCTCGGCCCTACATGG	CACAGAGTCGTCATCCGTCAC
aP2	ACACCGAGATTTCCTTCAAACTG	CCATCTAGGGTTATGATGCTCTTCA
Atgl	AACACCAGCATCCAGTTCAA	GGTTCAGTAGGCCATTCCTC
Cd36	TTGGCCAAGCTATTGCGACA	GCAAAGGCATTGGCTGGAAG
C/ebpalpha	GGACAAGAACAGCAACGAGTA	GCAGTTGCCCATGGCCTTGA
C/ebpbeta	TGGACAAGCTGAGCGACGAG	TGTGCTGCGTCTCCAGGTTG
CGI-58	TGGTGTCCCACATCTACATCA	CAGCGTCCATATTCTGTTTCCA
Cpt1a	TGCACTACGGAGTCCTGCAA	GGACAACCTCCATGGCTCAG
Cpt2	CAACTCGTATACCCAAACCCAGTC	GTTCCCATCTTGATCGAGGACATC
Csf1r	GCATACAGCATTACAACTGGACCTACC	CAGGACATCAGAGCCATTCACAG
Emr1	CTTTGGCTATGGGCTTCCAGTC	GCAAGGAGGACAGAGTTTATCGTG
Fasn	CTGACTCGGCTACTGACACG	TGAGCTGGGTTAGGGTAGGA
G0s2	GGGAAGCTAGTGAAGCTATACG	CTGCACACCGTCTCAACTA
Hsl	GGCTCACAGTTACCATCTCACC	GAGTACCTTGCTGTCCTGTCC
Leptin	CAAGCAGTGCCTATCCAGA	AAGCCCAGGAATGAAGTCCA
Phlpp1	AGGGTCCCGGAGACGATAAG	AGGGCGGAGATGTCTTTTGC
Phlpp2	GGGCTGAGCGCCTCGTTGTT	ACGCCTGCCGTTGCCATCTC
Pparalpha	GGGTACCACTACGGAGTTCACG	CAGACAGGCACTTGTGAAAACG
Ppargamma	GTGCCAGTTTCGATCCGTAGA	GGCCAGCATCGTGTAGATGA
Scd1	CTCCTGCTGATGTGCTTCAT	AGGGTGCTAACGAACAGGCT
Srebp1c	GAAGCTGTCGGGGTAGCGTCT	CTCTCAGGAGAGTTGGCACCTG
