# Supplementary material for: Adipocyte PHLPP2 inhibition prevents obesity-induced fatty liver
Source: Nat Commun. 2021 Mar 23;12:1822. doi: 10.1038/s41467-021-22106-2 (PMC7988046; doi:10.1038/s41467-021-22106-2)
Supplement: Supplementary file 3 — Reporting Summary [file 41467_2021_22106_MOESM3_ESM.pdf]

nature research

Corresponding author(s): KyeongJin Kim, Utpal B. Pajvani

Last updated by author(s): Jan 5, 2021

Reporting Summary

Nature Research wishes to improve the reproducibility of the work that we publish. This form provides structure for consistency and transparency in reporting. For further information on Nature Research policies, see our Editorial Policies and the Editorial Policy Checklist.

Statistics

For all statistical analyses, confirm that the following items are present in the figure legend, table legend, main text, or Methods section.

n/a	Confirmed	
☐	☒	The exact sample size (n) for each experimental group/condition, given as a discrete number and unit of measurement
☐	☒	A statement on whether measurements were taken from distinct samples or whether the same sample was measured repeatedly
☐	☒	The statistical test(s) used AND whether they are one- or two-sided<br>*Only common tests should be described solely by name; describe more complex techniques in the Methods section.*
☐	☒	A description of all covariates tested
☒	☐	A description of any assumptions or corrections, such as tests of normality and adjustment for multiple comparisons
☐	☒	A full description of the statistical parameters including central tendency (e.g. means) or other basic estimates (e.g. regression coefficient) AND variation (e.g. standard deviation) or associated estimates of uncertainty (e.g. confidence intervals)
☐	☒	For null hypothesis testing, the test statistic (e.g. F, t, r) with confidence intervals, effect sizes, degrees of freedom and P value noted<br>*Give P values as exact values whenever suitable.*
☒	☐	For Bayesian analysis, information on the choice of priors and Markov chain Monte Carlo settings
☒	☐	For hierarchical and complex designs, identification of the appropriate level for tests and full reporting of outcomes
☒	☐	Estimates of effect sizes (e.g. Cohen's d, Pearson's r), indicating how they were calculated

Our web collection on statistics for biologists contains articles on many of the points above.

Software and code

Policy information about availability of computer code

Data collection	The confocal images were acquired by using Zen2 software.
Data analysis	Stastistical analysis: Microsoft excel 2016 or GraphPad Prism 9.0; Image analysis: ImageJ 1.52 software

For manuscripts utilizing custom algorithms or software that are central to the research but not yet described in published literature, software must be made available to editors and reviewers. We strongly encourage code deposition in a community repository (e.g. GitHub). See the Nature Research guidelines for submitting code & software for further information.

Data

Policy information about availability of data

All manuscripts must include a data availability statement. This statement should provide the following information, where applicable:

- Accession codes, unique identifiers, or web links for publicly available datasets
- A list of figures that have associated raw data
- A description of any restrictions on data availability

All data supporting the findings of this study are available from the corresponding authors on reasonable request.

Field-specific reporting

Life sciences study design

All studies must disclose on these points even when the disclosure is negative.

Sample size	No statistical methods were used to predetermine samples size. Sample size was chosen based on a previous publication (Kim K et al., Cell Metabolism 2018; 27(4): 816-827.e4), and the sizes are large enough to determine statistically significant effects.
Data exclusions	No data was excluded from this study.
Replication	Experimental findings were reliably reproduced in two to four times independent experiments as indicated throughout the manuscript.
Randomization	No particular procedure was applied for randomization since there were no experimental groups in this study.
Blinding	Investigators were not blinded during data collection and analysis. The investigators did not consider blinding necessary because all groups, including cellular experiments, were treated the same way. Confocal images were taken by a blinded scientist.

Reporting for specific materials, systems and methods

We require information from authors about some types of materials, experimental systems and methods used in many studies. Here, indicate whether each material, system or method listed is relevant to your study. If you are not sure if a list item applies to your research, read the appropriate section before selecting a response.

Materials & experimental systems

n/a	Involved in the study
☐	☒ Antibodies
☐	☒ Eukaryotic cell lines
☒	☐ Palaeontology and archaeology
☐	☒ Animals and other organisms
☒	☐ Human research participants
☒	☐ Clinical data
☒	☐ Dual use research of concern

Methods

n/a	Involved in the study
☒	☐ ChIP-seq
☒	☐ Flow cytometry
☒	☐ MRI-based neuroimaging

Antibodies

Antibodies used	Immunoblots: antibodies against HSL (#4107 or #18381), p-HSL (S563) (#4139), p-HSL (S565) (#4137), p-HSL (S660) (#4126), Perilipin-1 (#9349), p-AMPKalpha (T172) (#2535), AMPKalpha (#2532), p-CREB (S133) (#9198), CREB (#9197), p-(Ser/Thr) PKA substrate (#9621), HA-tag (#3724), Adiponectin (#2789) and beta-actin (#4970) from Cell Signaling (Denvers, MA); PHLPP1, (A300-660A), PHLPP2 (A300-661A) from Bethyl Laboratories, Inc (Montgomery, TX); PPARalpha (SC-9000) from Santa Cruz Biotechnology (Dallas, TX).<br>Neutralization: adiponectin antibody (ab3455) from Abcam.<br>Immunofluorescence: antibody against HSL (#18381) from Cell Signaling (Denvers, MA).
Validation	All the antibodies used in this study are commercially available and validated for their use as indicated according to the manufacturer's website.

Eukaryotic cell lines

Policy information about cell lines

Cell line source(s)	3T3-L1 and 293T Cell lines have been obtained from the certified vendor, ATCC.
Authentication	Noe of the lines used were authenticated
Mycoplasma contamination	Cell lines used in this study were negative to mycoplasma by observation by fluorescence microscopy.
Commonly misidentified lines (See ICLAC register)	No commonly misidentified cell lines were used in this study.

Animals and other organisms

Policy information about studies involving animals; ARRIVE guidelines recommended for reporting animal research

Laboratory animals	Description of research mice used for experiments can be found in the relevant figure legends and Methods. 7 to 8-week-old male Cre- control and A-PHLPP2 mice (C57BL/6 background) were housed in a standard cage under temperature (22°C) and humidity (40-60%) controlled conditions with 12/12 h light/dark cycle.
Wild animals	No wild animals were used in this study
Field-collected samples	The study did not involve samples collected from the field
Ethics oversight	Mice were housed according to protocols approved by the Columbia University Institutional Animal Care and Utilization Committee.

Note that full information on the approval of the study protocol must also be provided in the manuscript.
